# Secondhand smoke exposure and risk of incident peripheral arterial disease and mortality: a Scotland-wide retrospective cohort study of 4045 non-smokers with cotinine measurement

**DOI:** 10.1186/s12889-018-5227-x

**Published:** 2018-03-19

**Authors:** Liya Lu, Daniel F. Mackay, Jill P. Pell

**Affiliations:** Institute of Health and Wellbeing, University of Glasgow, 1 Lilybank Gardens, Glasgow, G12 8RZ UK

**Keywords:** Peripheral arterial disease, Mortality, Secondhand smoke, Tobacco smoking, Cotinine

## Abstract

**Background:**

Active smoking is an important risk factor for all-cause mortality and peripheral arterial disease (PAD). In contrast, published studies on the associations with secondhand smoke (SHS) are limited. The aim of this study was to examine the associations between SHS exposure and incident PAD, as well as mortality, among middle-aged non-smokers.

**Methods:**

We undertook a retrospective, cohort study using record linkage of the Scottish Health Surveys between 1998 and 2010 to hospital admissions and death certificates. Inclusion was restricted to participants aged > 45 years. Cox proportional hazard models were used to examine the association between SHS exposure and incident PAD (hospital admission or death) and all-cause mortality, with adjustment for potential confounders.

**Results:**

Of the 4045 confirmed non-smokers (self-reported non-smokers with salivary cotinine concentrations < 15 ng/mL), 1163 (28.8%) had either moderate or high exposure to SHS at baseline. In men, high exposure to SHS (cotinine ≥2.7 ng/mL) was associated with increased risk of all-cause mortality (fully adjusted hazard ratio [HR] 1.54, 95% CI 1.07–2.22, *p* = 0.020) with evidence of a dose-relationship (p for trend = 0.004). In men, high exposure to SHS was associated with increased risk of incident PAD over the first five years of follow-up (fully adjusted HR 4.29, 95% CI 1.14–16.10, *p* = 0.031) but the association became non-significant over longer term follow-up.

**Conclusions:**

SHS exposure was independently associated with all-cause mortality and may be associated with PAD, but larger studies, or meta-analyses, are required to confirm the latter.

## Background

Secondhand smoke (SHS), also called ‘environmental tobacco smoke’ or ‘passive smoking’ or ‘involuntary smoking’, is the inhalation of tobacco smoke by people other than the active smoker. Worldwide, only 16% of the global population is covered by smoke-free legislation protecting them from exposure to SHS in indoor public and work places [1]. Legislation protecting against exposure in private vehicles has been introduced in a small number of countries but protects children only, and non-smokers are not protected by legislation from exposure in their own homes. In Scotland, six years after the implementation of the smoke-free legislation banning smoking in enclosed public places, 17% of non-smoking adults reported exposure to SHS in their home or someone else’s home and 11% reported exposure outside of buildings (http://www.gov.scot/Publications/2013/09/3684/8). Active smoking is a well-established important and modifiable risk factor for all-cause mortality and atherosclerotic diseases including peripheral arterial disease (PAD) [2–4]. There is now substantial evidence that exposure to SHS also increases the risk of premature death [5], coronary heart disease [6] and stroke [7].

SHS is a mixture of air-diluted side-stream smoke from the burning cigarette tip, and ‘mainstream’ smoke exhaled by the smoker. Side-stream smoke contains higher concentrations of toxic gases and fine, respirable particles (< 2.5 μm diameters) than mainstream smoke [8–11]. Even short exposure to SHS has been demonstrated to be associated with impaired endothelium-dependent vasodilation in coronary arteries in non-smokers; comparable to habitual active smokers [12]. One hour exposure increases the concentrations of 11-dehydrothromoboxane B_2_ (11-DH-TXB_2_) and malondialdehyde (MDA) to those observed in active smokers [13]. A meta-analysis of 10 prospective cohort studies and 8 case-control studies, published in 1999, reported a pooled relative risk (RR) of 1.25 (95% CI 1.17–1.32) for coronary heart disease. There were clear dose relationships with increasing dose and duration of exposure to SHS from 1 to 19 cigarettes per day (RR 1.23, 95% CI 1.13–1.34) to more than 20 cigarettes per day (RR 1.31, 95% CI 1.21–1.42) and from 1 to 9 years of exposure (RR 1.18, 95% CI 0.98–1.42), and 10–19 years of exposure (RR 1.31, 95% CI 1.11–1.55) to more than 20 years of exposure (RR 1.29, 95% CI 1.16–1.43) [6]. A subsequent meta-analysis, published in 2011, of 4 cross-sectional, 6 case-control, and 10 cohort studies, showed an increased risk of stroke among non-smokers who were exposed to SHS (RR 1.25 95% CI 1.12–1.38), with a dose relationship whereby the risk increased from exposure to 5 (RR 1.16, 95% CI 1.06–1.27) to 40 (RR 1.56, 95% CI 1.25–1.96) cigarettes per day [7].

In contrast, only five published cross-sectional studies have examined the association between SHS exposure and PAD [14–18]. Three of these studies ascertained SHS exposure based on self-report [15, 17, 18] and the other two measured cotinine concentration [14, 16]. Cotinine, a nicotine metabolite, is an objective measure of tobacco exposure from all sources [19]. Four of the studies demonstrated an overall association [15–18], but one showed an association with PAD for only very high levels of exposure to SHS (cotinine > 155 ng/mL) [14]. To date, there have been no cohort studies published that have examined the association between SHS exposure in non-smokers and incident PAD. Therefore, we linked individual-level data from the Scottish Health Surveys to subsequent health records to examine whether SHS exposure, measured by salivary cotinine concentration, was an independent risk factor for incident PAD, as well as all-cause mortality, among non-smokers.

## Methods

### Data sources

The Scottish Health Surveys (SHeSs) are cross-sectional studies designed to gain knowledge about the health of the residents of private households across Scotland (http://www.scotland.gov.uk/Topics/Statistics/Browse/Health/scottish-health-survey). The Survey was first conducted in 1995, with subsequent surveys undertaken in 1998 and 2003 and then annually since 2008. The Surveys use a multistage, stratified probability sampling frame and different households were recruited into each Survey. Household response rates were 81% in 1995, 76% in 1998, 68% in 2003, and 61%–64% between 2008 and 2010 [20]. The methodology has been described in detail previously [16, 21]. Briefly, a two-stage process was used: a face-to-face interview was conducted by trained staff during which they collected information on demographics (including age, sex, postcode of residence, education, employment and income) and lifestyle (including alcohol intake, physical activity and smoking status) via computer assisted personal interviewing (CAPI), followed by a visit by a nurse who collected measurements (including height, weight and blood pressure) using standard operating procedures. All individuals aged ≥16 years were invited to provide a saliva sample for cotinine assay and a blood sample for assays including lipid concentrations. In each survey, over 90% of the participants consented to passive follow-up via record linkage to routine administrative data [20]. In Scotland, the Information Services Division (ISD) of the National Health Service (NHS) links, at an individual level, several Scotland-wide databases including death certificates (collated by the General Registrar Office) and admissions to acute hospitals (Scottish Morbidity Record SMR01).

### Inclusion criteria and definitions

In this study, we combined the 1998, 2003, 2008 and 2010 Surveys as they provided consistent information on salivary cotinine and diagnosis of PAD at baseline. Our study was restricted to participants aged > 45 years old who classified themselves as non (never or ex) smokers and whose salivary cotinine concentration was < 15.0 ng/ml [22]. Individuals with PAD at baseline and those who reported taking nicotine replacement products were excluded. PAD at baseline was defined as intermittent claudication ascertained using the Edinburgh Claudication Questionnaire [23]. SHS exposure was categorised into low (cotinine < 0.7 ng/mL), moderate (cotinine 0.7–2.6 ng/mL) and high (cotinine 2.7–14.9 ng/mL) [16, 21]. In Scotland, area-based deprivation is assessed using the Scottish Index of Multiple Deprivation (SIMD) which is derived from information on income, employment, health, education, housing, crime and access to services. The SIMD is used to derive quintiles of deprivation, ranging from 1 (most deprived) to 5 (least deprived) for the general Scottish population (http://www.scotland.gov.gov.uk/Topics/Statistics/SIMD). The study participants were categorised into these quintiles based on their postcode of residence. Body mass index (BMI) was classified as underweight / normal weight (< 25 kg/m^2^), overweight (25–30 kg/m^2^) and obese (≥30 kg/m^2^) [24]. Physically active was defined as self-report of any kind of physical activity for at least 3 h per week. Alcohol consumption was self-reported as: never drinker, ex drinker, low-risk drinker (men < 28 units/week; women < 21 units/week), increasing-risk drinker (men < 50 units/week; women < 35 units/week) and high-risk drinker (men ≥50 units/week; women ≥35 units/week). We used record linkage to SMR01 records and death certificates to identify the first hospital admission/death following the Survey in which PAD was recorded as the primary or secondary cause, defined as: International Classification of Disease, Tenth Version (ICD-10) A48.0, I10.5, I73.9, I70.2, I70.9, I74.3, I74.5, I79.2, R02; the International Classification of Disease, Ninth Version (ICD-9) 250.7, 440.20, 440.21, 440.22, 440.23, 440.24, 440.29, 443.9, 443.81, 707.10, 785.4; or Office of Population Censuses and Surveys Classification of Surgical Operations and Procedures (OPCS) X09.3, X09.4, X09.5, X09.8, X09, X10.1, X10.4, X10.8, X10.9, X11.1, X11.2, X11.8, X11.9, X12.1, L54.1, L63.1. Audits have shown that the SMR data are around 99% complete and over 90% accurate [20]. The linked data provided follow-up information to 31 December 2011.

### Statistical analyses

Differences in baseline characteristics by SHS exposure were summarised using chi-square tests for categorical variables and chi-square tests for trend for ordinal variables. Incident PAD cases were ascertained based on hospital admissions or deaths due to PAD. We used person-time incidence rate which can account for participants who entered the study at different times and those who were lost to follow-up or died during the study period [25]. The numerator was the number of incident PAD cases and the denominator was the sum of all of the person-years of observation for all study participants. Tests of Cox proportional-hazards assumptions were performed using Stata estat phtest [26]. Separate Cox proportional hazard models were developed to examine the association between levels of SHS exposure and two separate outcomes: incident PAD (hospital admission or death) and all-cause mortality. We ran several models with increasing levels of statistical adjustment for potential confounders: unadjusted, partially adjusted (age and sex) and fully adjusted (partially adjusted model plus deprivation quintile, BMI category, physical activity, alcohol consumption and survey year) using cotinine < 0.7 ng/mL as the reference category [16]. Statistical interactions with covariates were tested using likelihood ratio tests [27, 28]. Statistical significance was defined as a two-sided *p*-value < 0.05 for both main effects and interactions. The probability of survival to a given time point was estimated using the Kaplan-Meier method. The cumulative hazard of PAD over time was estimated using the Nelson-Aalen method. The log-rank test was used to assess if the curves representing different groups, in this case, different SHS exposure groups, differed significantly. All statistical analyses were performed using Stata 12.0 (Stata Corporation, College Station, Texas, USA).

## Results

**Fig. 1:**
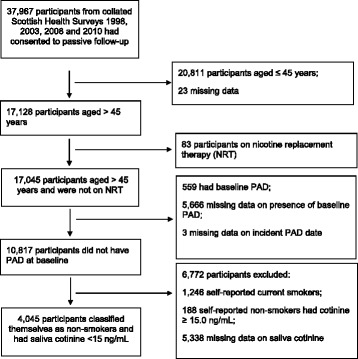
Flow diagram of participant inclusion and exclusion

Of the 41,664 participants in the four Scottish Health Surveys, 37,967 (91.1%) had consented to passive follow-up via record linkage to routine administrative data. Among these, 17,128 (45.1%) were aged > 45 years. Of these, 83 were excluded because they were on nicotine replacement therapy (NRT). Of the remainder, 10,817 participants completed the Edinburgh Claudication Questionnaire and were free of intermittent claudication. Of these participants, 6772 were excluded because: 1246 reported being current smokers, 188 reported being non-smokers but had a cotinine concentration ≥ 15 ng/mL, and 5338 did not provide a saliva sample. Therefore, 4045 participants comprised this study population (Fig. 1).

Of these, 2216 (54.8%) classified themselves as never smokers and 1829 (45.2%) as ex-smokers. Among ex-smokers, 1774 (97.0%) had quit smoking at least one year prior to the survey and 1620 (88.6%) at least five years. Overall, 1163 (28.8%) participants had either moderate or high exposure to SHS at baseline. The mean age at recruitment was 61 (SD 10) years and there was a total of 29,040 person years of follow-up (mean follow-up 9 years). Over the follow-up period there were 568 deaths, none of which were coded as due to PAD, and 64 people were hospitalised for PAD. All-cause mortality rates were 18.1% (95% CI 16.3%–20.0%) per 1000 person-years, 23.0% (95% CI 19.5%–27.0%) per 1000 person-years, and 25.3% (95% CI 19.7%–32.6%) per 1000 person-years among participants with no or low, moderate and high SHS exposure respectively. PAD incidence rates were 2.0% (95% CI 1.5%–2.7%) per 1000 person-years in participants with no or low SHS exposure, 2.5% (95% CI 1.5%–4.1%) per 1000 person-years in participants with moderate exposure, and 3.3% (95% CI 1.7%–6.2%) per 1000 person-years in participants with high exposure. Among male participants, PAD incidence rate was 5.7% (95% CI 2.7%–12.0%) among those with high exposure, compared with 1.5% (95% CI 0.6%–3.7%) among those with moderate exposure and 1.8% (95% CI 1.1%–3.0%) among those with low or no exposure.

**Fig. 2:**
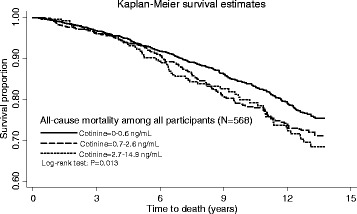
Survival proportion of all-cause mortality among all participants by cotinine concentrations using Kaplan-Meier method

Compared with the no or low SHS exposure group, participants with high exposure were older, and more likely to be male, obese and socioeconomically deprived; they drank more alcohol and were less physically active (Table 1). There was a statistically significant association between baseline exposure to SHS and all-cause mortality overall (Table 2, Fig. 2) and among the male sub-group of participants (Table 2). Figure 2 illustrates the difference across SHS exposure groups in terms of probability of survival (log-rank test *p* = 0.013). In the univariate and multivariate Cox proportional hazard models, there was a significant overall association between SHS exposure and mortality (Table 2). Among men, there was a clear, and significant, dose relationship that persisted in the age-adjusted and fully adjusted models (Table 2).

**Table 1:** Baseline characteristics of non-smokers by cotinine concentrations

	Cotinine (ng/mL)			
	0–0.6	0.7–2.6	2.7–14.9	
	*N* = 2882	*N* = 850	*N* = 313	*P* values^a^
	N (%)	N (%)	N (%)	
Age (years)				
45–59	1338 (46.4)	406 (47.8)	124 (39.6)	0.042
≥ 60	1544 (53.6)	444 (52.2)	189 (60.4)	
Sex				
Male	1250 (43.4)	423 (49.8)	167 (53.4)	< 0.001
Female	1632 (56.6)	427 (50.2)	146 (46.6)	
Deprivation quintile				
1(most deprived)	315 (10.9)	137 (16.1)	75 (24.0)	< 0.001
2	479 (16.7)	190 (22.4)	82 (26.2)	
3	622 (21.6)	202 (23.8)	55 (17.6)	
4	697 (24.2)	153 (18.0)	50 (16.0)	
5(least deprived)	644 (22.3)	141 (16.6)	40 (12.8)	
Missing	125	27	11	
Body mass index (kg/m^2^)				
< 25.0	670 (23.2)	151 (17.8)	46 (14.7)	< 0.001
25.0–29.9	1181 (41.0)	347 (40.8)	122 (39.1)	
≥ 30	757 (26.3)	272 (32.0)	122 (39.0)	
Missing	274	80	23	
Physically active				
No	1441 (50.0)	450 (52.9)	177 (56.5)	< 0.001
Yes	1251 (43.4)	332 (39.1)	101 (32.3)	
Missing	190	68	35	
Alcohol consumption				
Never drinker	237 (8.2)	51 (6.0)	25 (8.0)	< 0.001
Ex drinker	129 (4.5)	43 (5.1)	23 (7.3)	
Low-risk drinker	2300 (79.8)	655 (77.1)	211 (67.4)	
Increasing-risk drinker	163 (5.7)	66 (7.8)	33 (10.5)	
High-risk drinker	51 (1.8)	34 (4.0)	19 (6.1)	
Missing	2	1	2	
Smoking status				
Never smokers	1631 (56.6)	434 (51.1)	151 (48.2)	< 0.000^b^
Ex-smokers stopped smoking ≥5 years	1149 (39.9)	341 (40.1)	130 (41.5)	
Ex-smokers stopped smoking ≥1 year	1225 (42.5)	398 (46.8)	151 (48.2)	
Ex-smokers stopped smoking < 1 year	22 (0.8)	16 (1.9)	11 (3.5)	
Missing	4	2	0	

^a^χ^2^ test

^b^χ^2^ test for smoking status (never smokers, ex-smokers stop ≥1 year, ex-smokers stop < 1 year)

**Table 2:** Cox proportional hazard models of the association between secondhand smoke exposure, peripheral arterial disease and all-cause mortality

	Cotinine (ng/mL)	Number of events/ number of participants	Unadjusted			Partially adjusted†			Fully adjusted‡		
			HR (95%CI)	*P* value	*P* value for trend	HR (95%CI)	*P* value	*P* value for trend	HR (95%CI)	*P* value	*P* value for trend
PAD incidence											
All non-smokers^1^	0–0.6*	40/2882	1.00	–	0.172	1.00	–	0.140	1.00	–	0.382
	0.7–2.6	16/850	1.26 (0.71–2.25)	0.437		1.30 (0.73–2.33)	0.372		1.15 (0.64–2.06)	0.648	
	2.7–14.9	8/313	1.64 (0.77–3.49)	0.203		1.66 (0.77–3.51)	0.184		1.38 (0.65–2.95)	0.400	
Male non-smokers^1^	0–0.6*	16/1250	1.00	–	0.100	1.00	–	0.084	1.00	–	0.280
	0.7–2.6	5/423	0.82 (0.30–2.24)	0.702		0.91 (0.33–2.49)	0.848		0.76 (0.28–2.07)	0.595	
	2.7–14.9	7/167	2.89 (1.18–7.10)	0.021		2.82 (1.14–6.96)	0.024		2.10 (0.78–5.65)	0.141	
Female non-smokers^1^	0–0.6*	24/1632	1.00	–	–	1.00	–	–	1.00	–	–
	0.7–2.6	11/427	1.66 (0.81–3.38)	0.165		1.65 (0.81–3.37)	0.168		1.51 (0.73–3.15)	0.266	
	2.7–14.9	1/146	**	**		**	**		**	**	
All-cause mortality											
All non-smokers^1^	0–0.6*	362/2882	1.00	–	0.004	1.00	–	0.001	1.00	–	0.043
	0.7–2.6	145/850	1.25 (1.03–1.52)	0.022		1.34 (1.10–1.63)	0.003		1.24 (1.02–1.51)	0.034	
	2.7–14.9	61/313	1.30 (1.04–1.79)	0.024		1.42 (1.09–1.86)	0.011		1.21 (0.91–1.61)	0.194	
Male non-smokers^1^	0–0.6*	178/1250	1.00	–	0.006	1.00	–	0.001	1.00	–	0.004
	0.7–2.6	85/423	1.26 (0.98–1.63)	0.077		1.47 (1.13–1.92)	0.004		1.40 (1.07–1.83)	0.014	
	2.7–14.9	41/167	1.52 (1.09–2.13)	0.014		1.54 (1.08–2.18)	0.016		1.54 (1.07–2.22)	0.020	
Female non-smokers^2^	0–0.6*	184/1632	1.00	–	0.475	1.00	–	0.504	1.00	–	0.523
	0.7–2.6	60/427	1.14 (0.85–1.53)	0.380		1.14 (0.85–1.53)	0.368		1.03 (0.76–1.40)	0.836	
	2.7–14.9	20/146	1.07 (0.68–1.70)	0.764		1.05 (0.67–1.65)	0.828		0.80 (0.51–1.27)	0.344	

*HR* hazard ratio, *CI* confidence interval, *PAD* peripheral arterial disease

*reference; ** only one participant; † adjusted for age and sex for all non-smokers, adjusted for age for male or female non-smokers; ‡ partially adjusted plus deprivation quintile, body mass index, physical activity, alcohol consumption and survey year

^1^Test of proportional-hazards assumption all *p* ≥ 0.050

^2^Test of proportional-hazards assumption all *p* < 0.050

**Fig. 3:**
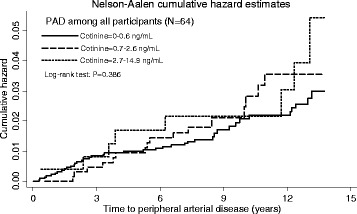
Cumulative hazard of peripheral arterial disease (PAD) among all participants by cotinine concentrations using the Nelson-Aalen method

**Fig. 4:**
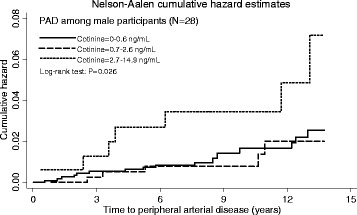
Cumulative hazard of peripheral arterial disease (PAD) among male participants by cotinine concentrations using the Nelson-Aalen method

In relation to incident PAD, the association with baseline exposure to SHS did not reach statistical significance overall (Table 2). There was no difference across SHS exposure groups overall (Fig. 3: log-rank test *p* = 0.386). However, there was a significant interaction with sex (*p* = 0.025). Male participants with high exposure to SHS were significantly more likely to experience PAD events in the unadjusted and age-adjusted models (Table 2, Fig. 4). Figure 4 illustrates the difference in the cumulative incidence of PAD across SHS exposure groups (log-rank test *p* = 0.026). After adjustment for other potential confounders, the hazard ratio was attenuated and no longer reached statistically significant. Among female non-smokers, there were no significant associations between baseline exposure to SHS and either all-cause mortality or PAD hospitalisations. There were no significant interactions with other covariates. The proportional hazards assumptions were met (global test: all *p* > 0.05) in all of the models except for the adjusted models for all-cause mortality in the female only sub-group (global test: *p* = 0.018 for partially adjusted model and *p* < 0.001 for fully adjusted model). The numbers of participants were too small to run subgroup piecewise analysis stratified by the other covariates.

Among male non-smokers, the association between high SHS exposure (cotinine ≥2.7 ng/mL) and incident PAD was significant over the first five years of follow-up even in the fully adjusted model (unadjusted HR 5.04, 95% CI 1.42–17.80, *p* = 0.012; fully adjusted HR 4.29, 95% CI 1.14–16.10, *p* = 0.031) but the association was weaker and became non-significant for events that occurred more than five years from baseline (unadjusted HR 1.83, 95% CI 0.50–6.75, *p* = 0.359; fully adjusted HR 1.34, 95% CI 0.28–6.54, *p* = 0.715).

## Discussion

Our study demonstrated that, among non-smokers, exposure to SHS was associated with an increased risk of all-cause mortality, with evidence of a dose relationship. Among male non-smokers, there was also evidence that high exposure to SHS (cotinine ≥2.7 ng/mL) was associated with increased risk of incident PAD over the subsequent five years.

Our finding of an independent association between SHS exposure and all-cause mortality is consistent with previous findings. Retrospective data from 192 countries were used to assess the global burden of disease and estimated that around 603,000 premature deaths in 2004 were attributable to SHS, with ischaemic heart disease making the largest contribution to SHS-related deaths [5].

PAD often co-exists with coronary heart disease and stroke in the same individuals [29]. They share many common risk factors including active cigarette smoking [2, 29]. Two meta-analyses have shown that exposure to SHS is associated with increased risk of coronary heart disease and stroke respectively [6, 7]. In contrast, so far, only five published individual studies have assessed SHS exposure and PAD, and all were cross-sectional in design [14–18]. Four of these reported significant associations [15–18]. One study was conducted among 1209 Chinese women aged ≥60 years who had never smoked. The study reported a significant overall association with PAD whether ascertained from the WHO Rose Questionnaire (adjusted OR 1.87, 95% CI 1.30–2.68, *p* = 0.001), ankle-brachial pressure index (ABPI) < 0.90 (adjusted OR 1.47, 95% CI 1.07–2.03, *p* = 0.018), or by either (adjusted OR 1.67, 95% CI 1.23–2.16, *p* < 0.001). There was evidence of a dose-response relationship in relation to both the number of cigarettes the participants were exposed to each day and the daily duration of exposure [15]. This was followed by a study which analysed data on 5653 non-smokers aged > 40 years in the USA using data from the National Health and Nutrition Examination Surveys. When non-smokers were dichotomised into those exposed to SHS (serum cotinine 0.05–10 ng/mL) and those not (cotinine < 0.05 ng/mL), there was no association with PAD, defined by ABPI. However, when SHS exposure was categorised into a larger number of sub-groups, the investigators reported a significant association with cotinine concentrations > 155 ng/mL and suggested a possible threshold effect [14]. Prior to this present study, we previously conducted two cross-sectional studies on the Scottish general population [16, 17] and one cross-sectional study on the Chinese general population [18]. Analysis of baseline data from the Scottish Family Health Study on 5686 never smokers aged ≥18 years demonstrated an overall association between self-reported level of SHS exposure and PAD defined by ABPI < 0.90 (adjusted OR 5.56, 95% CI 1.82–17.06, *p* = 0.003 for total exposure ≥40 h per week) [17]. The second study used baseline data from the Scottish Health Survey on 4231 non-smokers (self-reported non-smokers with salivary cotinine < 15 ng/mL) aged > 45 years. Participants with cotinine concentrations ≥2.7 ng/mL were significantly more likely to have intermittent claudication ascertained using the Edinburgh Claudication Questionnaire (adjusted OR 1.76, 95% CI 1.04–3.00, *p* = 0.040), compared to those with no or low SHS exposure (cotinine < 0.7 ng/mL). There was evidence of a dose-relationship across cotinine concentrations rather than a threshold effect, with differences achieving statistical significance above cotinine concentrations of only 2.7 ng/mL [16]. The third study used baseline data from the Guangzhou Biobank Cohort Study: Cardiovascular Disease Sub-cohort Study on 1507 non-smokers who aged ≥50 years. Exposure to SHS at home of ≥25 h per week was associated with PAD defined by ABPI < 0.90 (adjusted OR 7.86, 95% CI 2.00–30.95, *p* = 0.003), with evidence of a dose-response relationship [18]. However, prior to the current study, there have been no published studies of the association between SHS and incident PAD. In fact, very few studies on the general population have collected information on both SHS exposure and PAD.

The previous cross-sectional studies were unable to exclude the possibility of reverse causation. By using a cohort design, we were able to demonstrate a temporal relationship between SHS exposure and subsequent development of PAD. We observed statistical differences across no, low and high SHS exposure groups in terms of the probability of survival using a Kaplan-Meier plot and in terms of cumulative hazard of incident PAD using a Nelson-Aalen plot. Using Cox proportional hazard regression analyses, we demonstrated a higher hazard of death among participants with SHS exposure, compared with those without. However, the higher hazard of incident PAD reached statistical significance only among male participants with high SHS exposure. Our cohort study used record linkage of a pan-Scotland representative survey of the general population. Over 90% of the participants in the baseline surveys consented to passive follow-up via record linkage to routine administrative data on hospitalisation and death certification [20]. Researchers have no control over the completeness and accuracy of routine administrative data; however, the SMR02 dataset is subject to regular quality assurance checks (http://www.isdscotland.org/Products-and-Services/Data-Quality/).

The Scottish Health Surveys measured SHS exposure objectively using salivary cotinine concentration. This enabled us to exclude smoking deceivers by applying a maximum cut-off of 15 ng/mL for salivary cotinine concentrations; as validated by the Society for Research on Nicotine and Tobacco [22]. In order to maximise statistical power, we included ex-smokers as well as never smokers but the majority of ex-smokers (88.6%) had quit smoking at least five years prior to participating in the Survey. Each Survey collected baseline data on demographics and lifestyle. Therefore, in our statistical models, we were able to adjust for potential confounders such as age, sex, socioeconomic deprivation quintiles, BMI, physical activity and alcohol consumption. We were able to study two outcomes, incident PAD and all-cause mortality, the same cohort. Exposure to SHS is associated with diabetes [30–32], dyslipidaemia [33–35] and raised blood pressure [35–37], and some previous studies have adjusted for these covariates [14, 15]. These conditions are likely to lie on the causal pathway and, thus, are more likely to be mediators than confounders. Therefore, we did not include them in the model as this would have biased the results towards the null [38].

In line with previous studies on PAD, we only included participants who were > 45 years of age [16, 39]. The Surveys did not use an objective measure, such as ABPI, to ascertain prevalent PAD; however, PAD at baseline was defined via a widely used and well-validated questionnaire, the Edinburgh Claudication Questionnaire. Therefore, we were able to exclude participants with symptomatic PAD at baseline but not those with asymptomatic disease. Since ex-smokers are more likely to have developed intermittent claudication than never smokers [2]; they will have been disproportionately excluded. If the magnitude of the association between SHS and incident PAD varies by smoking status; this could potentially have introduced bias. Previous studies have suggested that the magnitude of the association with active smoking is greater for PAD than coronary heart disease [29, 40, 41]. Therefore, it might be hypothesised that this should also be true of SHS exposure. However, it would be inappropriate to compare our results directly with similar studies conducted on coronary heart disease since our ascertainment of incident PAD was limited to severe cases warranting hospitalisation.

The main limitation of this study was that the ascertainment of incident PAD was restricted to those participants who developed PAD of sufficient severity to warrant hospitalisation or predispose to death. The true incidence of PAD is likely to be higher than that reported in our study, and the association demonstrated between SHS exposure and severe PAD may reflect the combination of an association with overall incidence and an association with disease progression.

Another limitation of our study was the lack of serial information on smoking status and SHS exposure. Among men, the association between SHS exposure at baseline and PAD was significant for incident events that occurred within five years of baseline data collection but reduced in magnitude and became statistically non-significant thereafter. The latter is likely to reflect measurement error due to baseline SHS exposure no longer being a good measure of current SHS exposure; and the former may be a better estimate of the true magnitude of association between SHS exposure and PAD.

## Conclusion

Our study corroborates previous findings of an association between SHS exposure and all-cause mortality and is the first to demonstrate an association between SHS exposure and incident PAD. Our findings, in relation to PAD, only reached statistical significance in men and over the first five years of follow-up. Larger studies with serial measurements of exposure are required to properly evaluate the association in women and over longer follow-up. In comparison with coronary heart disease and stroke, recording of PAD has been neglected in large cohort studies; and future studies should address this deficit. Our findings add weight to the existing evidence that SHS is a threat to public health and measures should be taken to protect the general population from exposure.
